# Consumer Intention to Utilize an E-Commerce Platform for Imperfect Vegetables Based on Health-Consciousness

**DOI:** 10.3390/foods12061166

**Published:** 2023-03-09

**Authors:** Phaninee Naruetharadhol, Sasichakorn Wongsaichia, Teerapong Pienwisetkaew, Johannes Schrank, Kullanan Chaiwongjarat, Peeranut Thippawong, Thanaphat Khotsombat, Chavis Ketkaew

**Affiliations:** 1 International College, Khon Kaen University, Khon Kaen 40002, Thailand; 2 Center for Sustainable Innovation and Society, Khon Kaen University, Khon Kaen 40002, Thailand

**Keywords:** health-consciousness, imperfect fruit and vegetables, food delivery platform, organic food, young adult

## Abstract

Thailand has a problem with fruit and vegetable waste because consumers have a negative attitude towards fruit and vegetables with imperfect shapes; however, those imperfections have no impact on nutritional quality. Young adults are most aware of the problem of food waste. Here, we study factors influencing consumer’ intention to adopt an e-commerce platform that commercializes imperfect vegetables (ugly veggies). In all, 390 respondents from four provinces of Thailand were enrolled in the study. Cluster analysis, structural equation modeling, and multigroup moderation analysis approaches were used. The main conceptual framework was adapted from the unified theory of acceptance and the use of technology. Respondents were classified into low, moderate, and high health-conscious segments. We found that performance expectancy positively influences the behavioral intention of highly health-conscious consumers. For consumers in the moderate health-consciousness segment, effort expectancy and social influence positively affect behavioral intention. None of the hypothesized factors influence the low health-consciousness segment’s behavioral intention. This paper expands the understanding of consumer’ attitudes toward accepting an imperfect vegetable e-commerce platform. Additionally, the research can guide platform development and marketing efforts.

## 1. Introduction

The problem of food waste and food loss has been defined by the Food and Agriculture Organization of the United Nations [1]. Food waste is a reduction in the mass and nutritional value of food occurring in the production, harvesting, processing, distribution, sale, and consumption sectors within the food supply chain. Food waste is the loss of food at the end of the food chain during retail and consumption and is linked to the behavior of retailers and consumers [2]. Food waste in the fruit and vegetable sector is defined as fruit and vegetable waste (FVW), used with the inedible parts of fruit and vegetables that are discarded during collection, handling, transportation, and processing. This is sometimes called food loss [3]. Fruit and vegetable waste has long been recognized, yet consumers want to choose the best produce for themselves. Ultimately, FVW exists across the food supply chain, with around 40% of total FVW coming from grocery stores, contributing to global warming and our carbon footprint [4].

Because of their unattractive shapes, it is known that FVW is increasing, especially among farmers who have leftovers from vegetable production. When the appearance of the vegetables is unsatisfactory, they are often rejected at the farm level simply because they do not meet farmers’ standards, based on customer value and perception. Vegetable shapes do not affect taste or nutrition [5]. According to the 2021 World Population Data Sheet, it is estimated that by 2050 the world population will increase from 7.6 billion to 9.8 billion, and the world will need to increase food production by 56% to meet population demand (Population Reference Bureau (PRB)). The growing demand for food contrasts with the fact that every year the world produces more than 13 million tons of food waste, to which FVW contributes [6]. Fruit and vegetables are discarded upstream (e.g., at plantations), midstream (e.g., at product screening centers), and downstream (e.g., at modern and traditional trade retail chains) [7]. A recent US agricultural projection report noted that American farmers throw away up to 30% of their edible produce with imperfect shapes (~66.5 million tons annually) [8]. Over 25% of the world’s freshwater is wasted to grow those produce. In general, people have a negative perception of imperfect vegetables. A study regarding consumer behavior and unattractive produce found that people expected ugly produce to be less tasty and even less nutritious than attractive produce, even though there was no reason which would make it less delicious or less healthy [9]. It can be concluded that people use appearance to judge unattractive produce and make negative inferences.

With digital technology and e-commerce, there is a potential solution to solve food waste by reusing misshapen fruit and vegetables instead of discarding them. Consumers are increasingly using online services for convenience reasons, such as simple payment methods, and the size of their delivery networks have expanded [10]. Food delivery platforms can allow customers to access and order food easily by providing convenience, reducing waiting time, and recording customer preferences [11]. Supported by mobile devices [12], food delivery platforms can deliver high-quality, healthy food at a reasonable price. Moreover, selling ugly produce may offer customers products at relatively lower prices [13]. In 2014, French supermarkets began marketing ugly produce to promote the value of malformed food, leading the world’s food retailers to launch campaigns to change consumer’ perceptions of imperfect produce. Since then, more companies have started to sell ugly produce and use it in their products to prove that ugly food is edible, delicious, and nutritious.

While consuming ugly vegetables is new to Thailand, a similar concept already implemented in western countries could be viable. Therefore, this study presumes developing an online platform to be used as an e-commerce tool for farmers who want to sell their misshapen fruit and vegetables, and for consumers who accept the distortion and want to consume organic fruit and vegetables. The platform will use the concept of improving product value from imperfect produce and waste. The products are from imperfect fruit and vegetables, and from processed foods cooked from ugly produce. Several studies have addressed imperfect fruit and vegetables, including consumer’ perceptions, purchase behavior, motivations, and opinions [14,15]. Based on prior research, developing online platforms for selling ugly fruit and vegetables may be viable in Thailand.

Most research focuses on consumer’ behavior, acceptance, attitude, satisfaction, and innovation-adoption characteristics in attractive food products. In addition, the concept of ugly products is new to Thailand; there is a need to study market segmentations before developing marketing strategies. Categorizing consumers into segments helps marketers to understand consumer’ desires. However, few studies have focused on factors influencing behavioral intention (BI) and customer segmentation in ugly vegetable online platforms. Furthermore, this research adds to the existing literature by analyzing health-consciousness’s moderating role on several factors that impact behavioral intention. The paper classifies young adults as low, moderate, and high health-conscious consumers. The consumers are grouped based on their questionnaire responses regarding health-consciousness [16]. Young adults’ behavior and acceptance of imperfect vegetables may be affected by their degree of health-consciousness. Consumers with moderate and high health-consciousness might accept and use ugly veggies more easily than consumers with low health-consciousness. The impact of performance expectancy (PE), effort expectancy (EE), facilitating conditions (FCs), and social influence (SI) on BI may differ by the degree of health-consciousness. This paper investigates these moderating roles of health-consciousness.

Thus, this research aims to explore the significant relationship among factors such as PE, EE, SI, FCs, and BIs to use ugly vegetable e-commerce platforms. The factors are derived from the unified theory of acceptance and the use of technology (UTAUT) [17]. The study also aims to compare the differences between three consumer segments based on the level of health-consciousness. This study is interested in studying the attitude of young adults towards imperfect fruit and vegetables because digital technology may impact their food choice [18]. On the other hand, young adults have become more aware of the importance of organic food and the problem of food waste. Healthy food-consciousness is growing among young adults worldwide [4]. Hence, further studies and an analysis of the behavior of young adults toward accepting ugly veggies as healthy food may contribute to developing a viable ugly vegetable e-commerce platform.

## 2. Literature Review and Hypothesis Development

Several research articles related to the consumption of vegetables have used the structural equation model (SEM) to reveal the relationship between consumer’ perceptions and attitudes toward vegetables, marketing terms, and BI [14,15,19]. This study demonstrates consumer’ acceptance of an ugly vegetable e-commerce platform. The researchers utilized the theory of acceptance and the use of technology (UTAUT) to investigate how four factors (PE, EE, SI, and FCs) influence consumer’ BIs [17]. The relationship between variables was formulated and hypothesized using the SEM framework.

### 2.1. Development of the Unified Theory of Acceptance and the Use of Technology

Here, we investigate the factors that influence the acceptance and use of technology. The UTAUT was developed from eight theories on human behavior: the theory of reasoned action (TRA); the technology acceptance model (TAM); the motivational model (MM); the theory of planned behavior (TPB); combined TAM and TPB (C-TAM-TPB); the model of PC utilization (MPCU); innovation diffusion theory (IDT); and social cognitive theory (SCT) [17]. Venkatesh et al. [17] determined the purpose of creating UTAUT, applying dispersed theory and research on accepting individual information technology into a unified theory model [17]. Thus, the UTAUT is used to measure individual acceptance and gather common theoretical perspectives composed of four factors, as follows: PE, EE, SI, and FCs. The construction of this factor is causing the engender of dynamic influence, including age, gender, experience, and voluntariness of use. The UTAUT model can illustrate a technology acceptance potential of up to 70% [17]. According to the extended theoretical model, the perceived ease of use significantly impacts acceptance [20]. The customer-perceived value has a significant positive impact on adopting food-related digital technology, such as the food traceability system [21]. The UTAUT2 model analyzes customer’ intention to use online delivery services and studies essential factors that affect such intention [22]. The UTAUT model is appropriate for studying the acceptance of ugly vegetable e-commerce platforms.

#### 2.1.1. Performance Expectancy (PE)

Individuals believe using technology can increase efficiency in activities [17], reduce time duration, and gain productivity, including several factors such as perceived usefulness, outcome expectancy, job fit, long-term consequence, and relative advantage. Chan et al. [23] determined the PE factor, which studied technology acceptance and the diffusion of e-collaboration among SMEs. Their study was divided into three stages: evaluation available to use; the decision of use; and distribution to SMEs member. The PE factor influences technology acceptance with e-collaboration of three stages [23]. A previous article indicates that technology acceptance can increase efficiency and effectiveness in the working process, contributing to user’ willingness to use technology [24]. When the user interacts with a collaboration technology system, it is found that work efficiency helps increase user’ intention of the technology system [25]. A study using the UTAUT model for business purposes found that PE positively influences behavior’ intention to use this technology [11]. The research results demonstrate that trust is influenced by word of mouth of consumer’ satisfaction with shopping online. The quality of technology and information influenced PE and impacted the recommendation of this platform [26]. In addition, when people have a high-awareness of technology to contribute to performance in their work, they will have the intention to use technology [27], proving that the UTAUT theory is compatible with an ugly vegetable platform.

**Hypothesis** **1 (H1):** *Performance expectancy (PE) positively influences behavioral intention (BI) to use an ugly veggie platform*.

#### 2.1.2. Effort Expectancy (EE)

Effort expectancy is a belief among consumers that a technology system implementation does not require much effort [17]. According to the literature, EE is one of the most significant elements influencing technology adoption [28]. It involves perceptions of the ease of use, including perceptions that one can work through technology properly. Being able to learn, having good skills in working through technology, and recognizing work through technology is a simple process. EE is a significant variable that affects BI, generating the intention to utilize an online ugly veggies platform [12]. Allah Pitchay et al. [29] found that early in the behavior of new users, there are some obstacles associated with using technology. Once users are familiar with technology, that perception of the ease of use will also be more substantial. Overall, the willingness to use the technology was influenced by the perceived ease of use [22]. The ugly veggie platform will probably help simplify use and increase the demand of potential customers. A prior study using the UTAUT model with a new e-commerce platform indicated that EE slightly affected user’ BI to adapt to the platform [30]. Thus, it probably influences customer’ BI in using an ugly veggie platform.

**Hypothesis** **2 (H2):** *Effort expectancy (EE) positively influences behavioral intention (BI) to use an ugly veggies platform.*

#### 2.1.3. Facilitating Conditions (FCs)

A consumer’s degree of belief in enabling the use of technology is known as the facilitating conditions (FCs). The organizational structure that supports the use of technology entails acquiring the tools required for using the technology, or the always-on Internet network. Having the necessary knowledge and expertise to use technology and service personnel constantly available to assist customers when using technology is a challenge [17]. The facilitation condition represents the condition surrounding the person who can accommodate the customers of an ugly vegetable platform [31]. The FCs significantly affected the BI of users [32]. However, some research showed that mobile application implementation contributes to inducing healthy consumption and found that FCs do not impact consumer’ BI [19].

**Hypothesis** **3 (H3):** *Facilitating conditions (FCs) positively influences behavioral intention (BI) to use an ugly veggies platform.*

#### 2.1.4. Social Influence

Other people (e.g., family, partners, friends) can influence users. The SI refers to the potency of a colleague, employer, or close person, to influence the expression, action, or acceptance of new technology. In the early stage of developing technology, SI significantly affected user’ intention [17]. One UTAUT model and social network analysis study found that the recommendations of friends or influencers on the social network significantly impact consumer’ willingness to pay [20]. Thus, SI is important in influencing BI and decisions regarding technology acceptance [33]. A previous study believed that SI affects individual behavior, especially when society is important to the person [11]. This research found a relationship between social norms, perceived usefulness, and BI in e-learning technology, and indicated that SI significantly positively affects PE and behavioral intention [25]. Another study looked at the factors that affect a consumer’s intention to use an online platform for food delivery on a mobile phone, using the UTAUT model. This study found that SIs positively influence people’s feelings about online food delivery platforms. As a result, SIs positively affect attitudes toward an online food delivery platform [29].

**Hypothesis** **4 (H4):** *Social influence (SI) positively influences behavioral intention (BI) to use an ugly veggie platform.*

#### 2.1.5. Behavioral Intention

Behavioral intention (BI) represents people’s expectations and upcoming actions related to a specific behavior [34]. The BI in UTAUT predicts user’ intentions and behavior and correlates with actual usage [17]. It can be interpreted as the willingness to use a technology or system. It helps understand and predict adoption and usage behavior. Based on the UTAUT model, BI is directly influenced by PE, EE, and SI [17]. Further, BI is a major determinant predictor of actual use behavior [32]. Thus, BI is the key to developing the use behavior [35].

### 2.2. Moderating Roles of Health-Consciousness

Health-consciousness can be segmented as low, moderate, and high.

#### 2.2.1. High Health-Consciousness

Healthiness has become an important factor influencing people’s attitudes toward food consumption [36]. The degree to which individuals prefer to undertake, and are active in managing healthy behaviors, is referred to as health-consciousness [16]. It concerns how much health considerations are integrated into an individual’s daily routines [37]. The number of consumers concerned about their health continually increases [38]. People with high health-consciousness will be more concerned about their health and well-being [39]. Consumers with high health-consciousness are more likely to engage in healthy activities and healthy food to improve their health status [39]. Health-consciousness has become a major factor influencing the consumer’ purchase intention of organic food [40], positively affecting health-related Internet use [41].

A study implied that people aged 55 years or older tend to consume fruit and vegetables more than young adults aged 25–54 years [42]. The younger groups have a higher ability to access fruit and vegetables to support their consumption, such as reaching fruit and vegetable sources and preparing food [38]. In addition, Mustafa et al. [43] found the health-consciousness attitude is positively associated with intrinsic factors in technology adoption as long as consumers can assess the benefits of that product provided, related to perceived performance or PE. Interestingly, when it comes to consumer’ health, people do not consider economic factors, including FCs. Instead, they are impacted by social influencing and satisfaction [43]; this reveals that health-consciousness is not an important factor, but is a mediator influencing consumer’ adoption.

#### 2.2.2. Moderate Health-Consciousness

Moderate health-consciousness or indulgence refers to an individual food consumption behavior that is considered a lowered importance of the health meaning of food. It is a behavior driven by pleasure-seeking or convenience, such as consuming salty and sweet snacks and ready-made meals, eating on the go, and overeating [44]. This consumption behavior may drive poor nutrition and unhealthy eating habits. Although some foods seem full of fat and calories, some indulgence foods are healthy and benefit health, such as dark chocolate, butter, and full-fat yogurt [45]. Indulgent behavior is an attitude of people who allow themselves whatever they want with less consideration for their wellness. Food consumption could be both positive and negative for their health.

A previous study showed that the number of overweight adolescents (young adults) has increased, leading to physical health problems [36]. They were influenced to indulge by several factors, one of which was from parents, as they have less time to prepare nutritious meals [37]. At the same time, parenting with good preparation could positively impact a child’s health. The research paper found that fruit and vegetable intake was significantly related to parental modeling [40]. Parental consumption of fruit and vegetables was a predictor of their children’s fruit and vegetable intake [45]. Thus, parents impact their children’s eating behaviors as social learning is derived from observation.

#### 2.2.3. Low Health-Consciousness

Low health-consciousness is the behavior of less motivated individuals to consume healthy food [46], which increases the number of snacks and junk food products. Young adults tend to choose junk food over healthy food. Likewise, food characteristics for low health-conscious consumers are fast, convenient, accessible, and cheap [37]. A study found that consumers with low health-consciousness choose food products based on non-health-related attributes, such as reasonable price and taste. They also believe unhealthy food provide better taste [47].

A study found that, among young adults, junk food was the most advertised food product on social media [48]. Promoting junk food through advertising increased consumption among young adults by nearly 50% [37]. In Thailand, young adults eat unhealthily relative to older people [49]. Food characteristics for low health-conscious consumers include taste, freshness, safety, and low price. As healthiness is not an essential factor to consider in this consumer group, the price level is the main factor affecting the decision of low health-conscious customers [42].

Hence, this study also hypothesizes that health-consciousness (low, moderate, and high) moderates the structural relationship given by the UTAUT model. In other words, it can be said that health-consciousness is assumed to play an essential role in segmenting the behaviors of the users of an ugly veggies platform.

**Figure 1:**
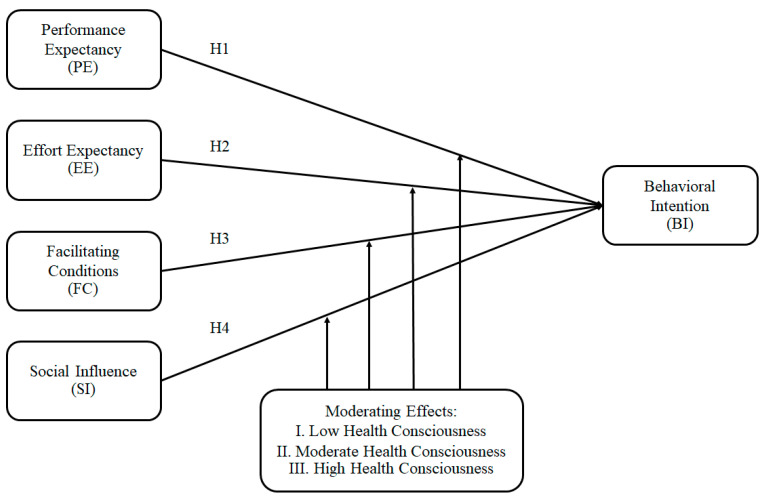
Proposed model. (Source: figure created by authors 2023).

Figure 1 illustrates the conceptual model of this study in light of the literature reviews and hypotheses.

## 3. Methodology

### 3.1. Pilot Test

The pilot test was performed to explore the existence of a market for health-conscious consumers. It was suggested to have a minimum sample size of 20–30 for a pilot study [50]. This study’s structure was investigated using a 5-point Likert scale. Due to its close location, we first decided to collect data from 100 respondents from Khon Kaen Province. The data from 100 respondents was used to test the demographic coverage, common method bias, and Cronbach’s alpha, in order to determine the viability of this study.

### 3.2. Sampling, Data Collection, and Measures

This research utilized a sampling method to collect data on Thai consumer’ intentions regarding imperfect vegetables. A questionnaire was used to collect data from a specific consumer segment determined by the researchers. (Appendix A) The questionnaires were collected using face to face surveys in the four northeastern provinces, with 100 respondents each: Khon Kaen (already collected from the pilot test), Nakhon Ratchasima, Kalasin, and Udon Thani. Purposive sampling was used because selecting specific samples was based on the rationality principle. A survey was used to collect data on customer acceptance of imperfectly appearing vegetables.

This research collected 400 participants from four provinces in the northeastern region, including Khon Kaen, Nakhon Ratchasima, Maha Sarakham, and Udon Thani, with 100 respondents from each province. The survey data collected likely locations for the health-conscious, including parks, fitness centers, and health food stores, and the non-health-conscious, including shopping malls, dessert shops, shabu/buffet restaurants, and markets. To ensure the respondent’s information remains confidential, data curation and utilization were based on the considerations for human research ethics (see the institutional review board statement section).

The questionnaire starts with a demographic profile including gender (male and female), marital status (single and married), family size (1, 2, 3, 4, >4), age (<25, 25–34), income (THB <10,000, 10,000–20,000, 20,001–30,000, 30,001–40,000, >40,000), educational level (Diploma or below, Bachelor, Master, Ph.D.), and occupation (student, government employee, state enterprise officer, employee, business owner). Next, the questions related to UTAUT were derived from the previous literature, for instance, PE [35,51,52], EE [35,51,52], FCs [35,51,52], SI [35,51,52], and BI [51,53] (Table 1). Researchers collected data about health attitudes and behavior to classify the consumer segments. Regarding health-consciousness as healthy actions, including consumer’ attitudes and health behavior, eight health-related questions were used to measure the consumer’ degree of health-consciousness. The questionnaires were implemented from previous research [54] (see Table 2). To prevent response bias, the questions were kept as short as possible and measured on a 5-point Likert scale with the following values: 1 = strongly disagree, 3 = neutral, and 5 = strongly agree [55]. Some items were coded to indicate that higher scores signify a higher level of health-consciousness [56]. High scoring represents well-being and a good health attitude, while low scoring represents lower health-consciousness [56]. In addition, the researchers only enable respondents to respond to the questionnaire once, to prevent ambiguity, which may lead to response bias.

### 3.3. Data Analysis

First, we tested a common method bias, a systematic error of variance that may exist through cross-sectional data collection. We used Harman’s single-factor test to examine this method bias. The test result is counted as 43.457%, and that value should not exceed the 50% threshold [57,58,59].

Next, using the cluster analysis technique, we classified the consumers into three segments based on the health-consciousness scores. According to the literature review, we utilized a hierarchical cluster analysis to separate respondents into three segments: high, moderate, and low heath-consciousness [37,39,44,45,46].

Then, we conducted mean comparisons using the health-consciousness scores of the three customer segments. First, normality and homogeneity tests were performed to meet the basic assumptions. We tested the data’s normality using Kolmogorov-Smirnov, Shapiro-Wilk, Skewness, and Kurtosis criteria. The test results revealed that the *p*-values from both Kolmogorov-Smirnov and Shapiro-Wilk tests were all statistically significant (*p*-value < 0.001). Further, the Skewness and Kurtosis values of the health-consciousness scores range from −0.915 to −0.178, properly falling into the threshold of −1.00 to 1.00. Hence, this data set satisfies the normality criteria. Next, the homogeneity test was performed using Levene statistics. The results showed that the *p*-values of five out of eight health-consciousness scores were not statistically significant, given a 5% significance level. This test indicated that the data set did not reveal equal means across three different groups, allowing us to use Welch’s ANOVA for mean comparisons in this research [60]. Welch’s ANOVA is often preferred over traditional one-way ANOVA because it is robust to differences in variance between groups. This is important because many real-world data sets do not meet the assumption of homogeneity of variance, and failing to account for this can result in inaccurate results. Using Welch’s ANOVA, this study provides a robust and accurate assessment of the differences in means between the groups [61,62,63]. Multiple comparisons were used to test whether the three populations’ mean differ [64].

Moreover, crosstabulation was used to demonstrate cluster results to demographic profile, and chi-square tests indicate a statistically significant result. This study investigated a model using the structural equation modeling (SEM) technique [65]. SPSS and AMOS statistical programs are instruments that contribute analysis data. The SEM is a statistical method for testing hypotheses and estimating causal relationships. The testing of the theory began with the formulation of a framework for testing hypotheses based on causal modeling; the data’s conformity was determined using confirmatory factor analysis (CFA). Commonly, the CFA is used as a confirmatory tool to determine whether a particular model is valid [66].

Moreover, this study used a multigroup analysis (MGA) to compare the likeness of the sample groups and analyze the data from each sample group at the time [66]. In each phase for evaluating SEM, the multigroup moderation analysis investigated the effects of segmentations on structural relationships. The selected segments are the results of the cluster analysis. Measurement invariance (MI) analysis was used to divide the sample into three groups: the high health-consciousness consumer segment, the moderate health-consciousness consumer segment, and the low health-consciousness consumer segment. The Z-test was used to examine the information of different segments.

## 4. Results

### 4.1. Step 1: Cluster Analysis

Most of the chi-square tests were significant, except for marital status (Table 3). Among the 109 highly health-conscious respondents (segment 1), 32 were male (8.20%), and 77 were female (19.70%). Most of these respondents have four people in their family (13.10%) and are 25–34 years old (young adult people) (18.70%). Their income is between THB 10,000–20,000 per month for 12.80%. This segment included employees and business owners.

The moderate health-conscious (segment 2) group consisted of 62 males (15.90%) and 112 females (28.70%). Most had a family size of three people (16.90%). They were mostly less than 25 years old (36.20%), with an income of THB 10,000–20,000 per month (25.60%), and holding a Bachelor’s degree (38.70%) or currently studying (40.08%).

Among the low health-conscious (segment 3), 65 were male (16.70%), 42 were female (10.80%), and 8.40% had three to four people in their family. This segment was mostly made up of young adults (18.70%) who had an income of THB 10,000–20,000 per month (18.70%) and were Bachelor students (29.50%). When employed, they were mostly office workers or government employees.

Based on questionnaire responses, we separated consumers into three groups: (1) high health-consciousness; (2) moderate health-consciousness; and (3) low health-consciousness. We performed Welch’s ANOVA to assess the mean differences of multisamples with unequal variances [67,68]. Table 4 demonstrates that the means of health-consciousness scores are different among the three customer segments (*p*-value < 0.001). The results imply that the cluster analysis can properly segment the sample consumers based on their level of health-consciousness. The means of health-consciousness scale scores of the high, moderate, and low health-conscious groups range between 4.20 and 4.38, 3.98 and 4.21, and 2.24 and 2.84, respectively. Explicitly, we found that consumers in segment 1 perceived that food influenced their health the most (mean = 4.44). Consumers in segment 2 likely think that a good knowledge of how to eat healthily is necessary (mean = 4.21). However, consumers in segment 3 have the impression that they sacrifice a lot for their health (mean = 2.84).

Next, we used the measurement model (CFA) and structural equation modeling (SEM) [69].

### 4.2. Step 2: Measurement Model

The measurement model was investigated using confirmatory factor analysis (CFA) to assess the reliability, internal consistency, discriminant, and convergent validity. It also proves a relationship between constructs, including the goodness of fit (GOF), average variance extracted (AVE), composite reliability (CR), and geterotrait-monotrait (HTMT) correlation ratio [70,71].

#### 4.2.1. The Goodness of Fit

The results show all the measures passed the required criterion (Table 5). Further, CMIN/df (1.739), the Tucker-Lewis sustainability index (TLI; 0.967), the comparative fit index (CFI; 0.974), the incremental fit index (IFI; 0.974), and the root mean square error of approximation (RMSEA; 0.044) passed the thresholds.

#### 4.2.2. Convergent Validity

This measurement model evaluates a structure result with a fit index criterion. We perform average variance extracted (AVE) to measure the variation collected by a construct relative to the variance attributable to measurement error [72]. Composite reliability (CR) defines the dependability and internal consistency of a construct’s indications [73] and Cronbach’s alpha checks to evaluate whether multiple-question Likert scale surveys are trustworthy [74]. The criteria is that AVE should be above 0.50, CR should be greater than 0.70, and the loading value of each construct should be more than 0.6, which indicates a high convergent validity [70,71]. As a result, the factors PE, EE, FCs, and SI passed the convergent validity index criterion (Table 6).

#### 4.2.3. Discriminant Validity

Discriminant validity refers to the extent to which similar constructs differ empirically. It can be evaluated by the cross-loading indicator, the Fornell and Larcker criterion, and heterotrait-monotrait (HTMT) correlation ratio. The HTMT criterion is a stringent measure that can detect possible discriminants among variables, while the Fornell and Larcker criterion can only detect small differences [75]. The HTMT criterion can detect collinearity issues among constructs. It has been suggested that the HTMT criterion is more sensitive and specific for detecting discriminant validity problems (97–99%) than the Fornell and Larcker criterion (20.82%) [75]. Thus, the HTMT criterion should be used to prevent misleading interpretations in modeling analysis and was chosen in this study.

The HTMT value of each latent construct should not be greater than 0.85. If the HTMT value is greater than 0.85, there is a problem with discriminant validity [66]. All HTMT values are lower than the required value of HTMT 0.85, which indicates that discriminant validity is valid (Table 7).

### 4.3. Step 3: Structural Equation Modelling

#### 4.3.1. Primary Structural Model

The constructs are connected to develop the structural model according to the proposed model in Figure 1. The goodness of fit (GOF) was developed as an overall measure of model fit for SEM. The GOF results show that the constructs support each other (see Table 8).

#### 4.3.2. The Goodness of Fit of the Structural Model

After validating the measurement model, the structural model was analyzed. First, the GOF indices passed all the thresholds (Table 8).

**Figure 2:**
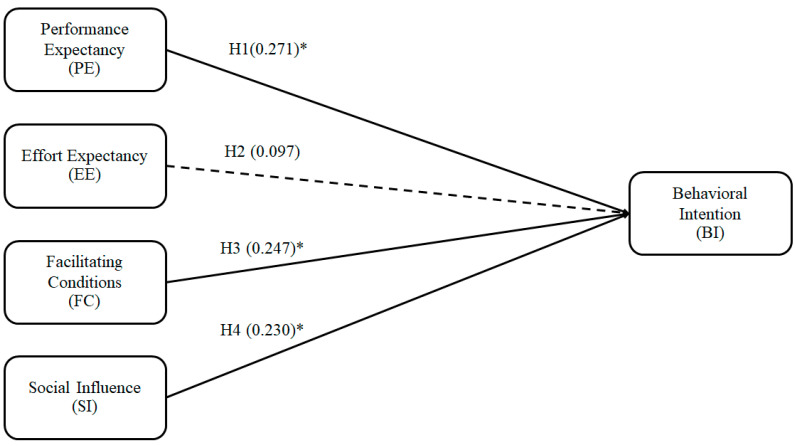
Structural model. Note: * Significance < 0.05(Source: figure created by authors 2023).

The findings from Table 9 and Figure 2 support the proposed model and hypotheses, demonstrating the influence of PE, FCs, and SI on BI. More explicitly, PE, FCs, and SI associate positively with BI (supporting H1, H3, and H4). However, EE does not significantly impact BI (rejecting H2).

Our findings suggest that young adults prefer to use platforms with high technical performance. Their intention to adopt technology increases when it can improve their lifestyles by providing convenience and efficiency [26,30,76,77]. The results also suggest that consumer’ decision-making is influenced by the people around them and social influences [20,29,30,33]. Additionally, the facilitating conditions significantly impacts the BI of consumers. It can be stated that consumers have concerns about the difficulties of using the platform as they have already experienced using other similar platforms [25]. However, H2 was rejected. Thus, EE does not influence the intention of adapting technology, as customers do not want to put much effort into using an ugly veggies platform [30].

### 4.4. Multigroup Moderation Analysis

#### 4.4.1. Measurement Invariance

Measurement invariance (MI) is a method that assesses whether the respondents between three groups (low health-consciousness, moderate health-consciousness, and high health-consciousness) are different [78]. We applied confirmatory factor analysis (CFA) to measure the information [79]. The MI is established when the values in Table 10 are satisfied, including configural invariance, metric invariance, and scalar invariance. There are differences (MI is supported) among the three segmentations when these values meet the standard.

The CMIN/df of the configural invariance, metric invariance, and scalar invariance passed the <3.00 threshold (Table 10). Other fit indexes were TLI, CFI, IFI (>0.90), and RMSEA (<0.10), passing the designated thresholds for configural, metric, and scalar invariances. When all invariance criteria are satisfied, the results are accounted as full measurement invariance. These findings enable us to conduct more analyses in the following parts.

The goodness of fit of the multigroup structural model is acceptable because the variable’s value meets the necessary thresholds (Table 11). The following criteria are: the Tucker Lewis index (TLI: 0.926); the comparative fit index (CFI: 0.938); the incremental fit index (IFI: 0.939); and the root mean square error of approximation (RMSEA:0.038).

#### 4.4.2. Z-Test for Loading Differences

The Z-test for loading differences is used to examine whether there are differences between the factor loadings (so-called loadings or standardized estimates) of the low, moderate, and high-conscious consumer groups. Loading difference refers to the difference in magnitude and direction between two loadings considering a particular path in the structural model. The loading reflects each variable’s contribution to the factors’ overall structure. Variables with high loadings on a particular path are considered more strongly associated with that factor, while variables with low loadings demonstrate a low association. When two groups’ loadings are significantly different (given a particular path), we can compare the strength of paths between the two groups.

**Figure 3:**
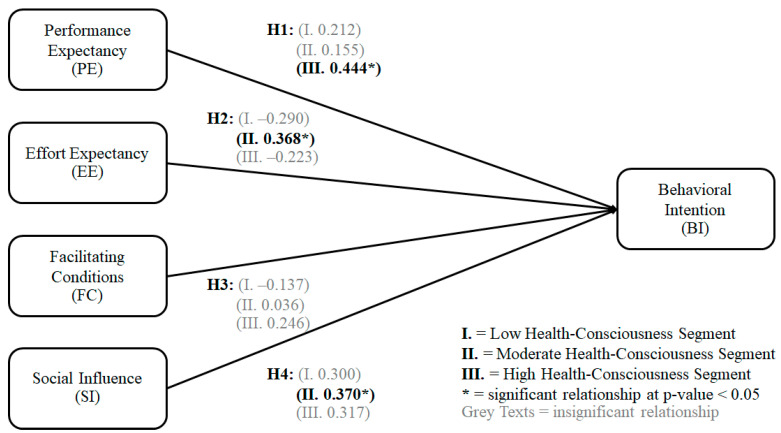
Multigroup structural model. (Source: figure created by authors 2023).

The researchers used the critical ratio difference as part of an MGA approach. If the loadings between the two factors are different, the critical ratio difference would be statistically significant. To evaluate, if the critical ratio is greater than the critical value for the specified level of significance, then the two observed factor loadings are considered to be significantly different [80]. To collect the Z-test results, the factor loadings of three groups were compared: (1) low vs. high health-consciousness; (2) low vs. moderate health-consciousness; and (3) high vs. moderate health-consciousness [66]. The critical ratio difference is greater than the threshold value of 1.96 (*p*-value = 0.05 for Z-test), indicating statistical significance. According to Table 12 and Figure 3, for H2, the critical ratio difference is greater than the threshold for high vs. moderate health-consciousness consumers (2.247 > 1.96), implying a difference between the loadings of these two groups. However, other critical ratio differences are not statistically significant because they do not exceed the threshold of |1.96|.

## 5. Discussion

### 5.1. High, Moderate, and Low Health-Consciousness Consumers

This section discusses the primary results of this research. Table 12 and Figure 3 show the hypothesis test results (H1, H2, H3, and H4) for multigroup analysis classified into three customer segments (I: low; II: moderate; and III: high health-consciousness). The figure shows that H3 has no statistically significant relationship with the BI for all three groups. PE positively influences the BI of highly health-conscious consumers (Std. Est. = 0.444). EE and SI positively impact the BI of moderate health-conscious consumers. The standardized estimates are 0.368 and 0.370, respectively. Low health-conscious consumers are not affected by any factors. Here, we deliberate the results in more detail.

The PE had a positive influence on the BI of the highly health-conscious consumers (Std. Est. = 0.444, *p* = 0.015). This explains that consumers with high health-consciousness assume that the technology can shorten the time it takes to use the technology and can fully support the user [32]. At the same time, consumers with low health-consciousness (Std. Est. = 0.212, *p* = 0.838) and moderate health-consciousness (Std. Est. = 0.155, *p* = 0.347) do not see the benefits of PE on the platform. This result confirms that consumers with high health-consciousness are more likely to participate in health-related activities, including adopting health technology [39,43,81]. Individuals prioritizing health and wellness tend to be most aware of these issues [39]. More health-consciousness among consumers predicts a greater uptake of preventative health measures, such as physical activity and dietary changes that benefit health [39]. A growing consumer’ concern for health has a favorable impact on interest in and willingness to utilize the Internet for information on health-related topics [40], leading consumers to be more likely to consider purchasing organic vegetables online. Increasing the efficiency of an e-commerce platform is essential. Technology adoption may improve workflow efficiency and user’ willingness to utilize technology [24]. Work efficiency increases the user’ intention of a collaborative technology system, especially for high health-conscious consumers [25].

Interestingly, EE positively affects BI for the moderate health-consciousness segment (Std. Est. = 0.368, *p* = 0.019). This can be explained by consumers who believe that implementing a technology system does not require much effort [32]. For the low health-conscious (Std. Est. = −0.290, *p* = 0.843) and high health-conscious consumers (Std. Est. = −0.233, *p* = 0.296), there was no statistically significant relationship with BI. Moderate health-conscious consumers anticipate technology system deployment to be easy [17], incorporating simplicity of use and technical proficiency. Further, EE influences BI and the inclination to use an online ugly veggies platform [12]. Technology will seem easier to use as consumers are accustomed to it. An ugly vegetable platform could simplify usage and boost the demand of the moderate heath-consciousness segment [22].

The facilitating conditions (FCs) did not positively affect BI in consumers with high health-consciousness (Std. Est. = 0.246, *p* = 0.417), low health-consciousness (Std. Est. = −0.137, *p* = 0.979), and moderate health-consciousness (Std. Est. = 0.036, *p* = 0.843). This is consistent with prior studies reporting that the facilitating condition is not a major factor in the adoption and use of technologies [19]. The MGA results imply that none of the consumer segments require facilitating conditions. When using health-consciousness as a criterion for consumer segmentation, it becomes clear that FCs do not accurately portray the online service settings that accommodate consumers of an ugly veggies platform [31]. The results are in line with a study that revealed that FCs had no effect on customer’ BI [19].

In addition, SI positively affected BI for moderate health-conscious consumers (Std. Est. = 0.370, *p* = 0.015). Using social network analysis, for the moderately health-conscious, it has been found that the recommendations of friends or influencers on social networks had a significant positive impact on consumer’ willingness to pay [20,81]. The low health-conscious (Std. Est. = 0.300, *p* = 0.891) and high health-conscious (Std. Est. = 0.317, *p* = 0.308) consumers do not have a statistically significant relationship with BI. The findings are consistent with the literature in that moderately health-conscious consumers need social influences to promote behavioral intention. People’s actions may be influenced by SI, according to previous research [11], particularly if they emphasize blending in with their peers. Findings from a similar research paper indicate that SI favorably impact people’s opinions of food delivery services that operate online. Hence, moderate health-conscious consumers require SI, which favorably affect how they feel about a service that provides online food deliveries [29].

### 5.2. General Discussions

Our results imply that PE impact consumer’ BI to use the ugly veggies platform. This is consistent with previous findings on organic food delivery technology [29]. It shows that consumers prefer using platforms with good technology effectiveness and efficiency. High technology awareness can affect consumer’ intention to use a platform [27]. The importance of PE in influencing BI has been reported [82]. We found that consumers with high health-consciousness are more concerned about the platform’s performance than other groups. According to their consuming lifestyle, they would like to use a platform that can deliver healthy products with high technology efficiency and ease of use.

However, we found that EE and FCs hurt consumer’ BI to adapt to the platform differently based on the health-consciousness segments [30]. We found that the consumers are not concerned about the difficulties of using platforms. This can be attributed to advancements in smartphones and technology; consumers expect to find it easy to use and adapt to the platform as they have experience using similar delivery apps [30]. Only young adults with moderate health-consciousness consider EE; their eating behavior is characterized as consuming anything they want, with little concern for healthiness [44]. We believe that effortlessness can influence their intention. Using the critical ratio difference (CRD), we detected a significant difference between high and moderate health-conscious groups when comparing the differences among three consumer groups. This implies that high health-conscious consumer’ intention does not depend on the platform’s ease of use, which contrasts with the moderate health-conscious that prefer using platforms requiring less effort and high accessibility. It has been reported that EE has a significant impact on consumer’ adoption intention for online food delivery platforms [25].

We detected a positive relationship between SI and behavioral intention, which aligns with a previous study [22,83]. Additionally, SI affects BI in adopting platforms [84]. We can conclude that young Thai adults are impacted by social life; the people around them easily influence them. Our result has interesting implications from this perspective. The finding implies that SI affects the BI of moderate health-conscious consumers. Friends and family likely affect the behavior of young Thai adults. Surprisingly, there were no such relationships among factors for the low health-conscious consumers. They do not consider any hypothesized determinants other than food taste and low price. According to their low income (THB 10,000–20,000), young consumers may consider purchasing costly organic food [85].

The finding regarding the lack of impact of PE, EE, FCs, and SI on the low health-conscious consumer’ behavioral intention to adopt the ugly veggies platform is crucial for understanding the consumer’ decision-making process in adopting e-commerce platforms for food delivery.

The results highlight the importance of considering the high and moderate health-conscious consumers in predicting their intention to use the platform. Moreover, it provides valuable insights for the platform providers to tailor their offerings and marketing strategies to cater to the needs of different consumer segments. By understanding the influence of various factors on consumer’ behavioral intentions, platform providers can optimize their services and increase the adoption of their platforms. This finding has significant practical implications for the food delivery industry and can be used to inform future research in this area.

## 6. Research Implication

This study investigates the possibility of consumers eating imperfect vegetables via the ugly veggie platform. This study benefits technology developers by helping them better understand consumer’ BIs. Our findings suggest that developers should focus on PE and FCs. The developers can apply these findings to create a platform that meets consumer’ demands. For example, a programmer can write code to develop the platform’s performance, such as code to manage to wait time, and make the platform available for every device. Furthermore, platform designers can design a template that is easy to use.

Additionally, marketers benefit by understanding consumer’ perspectives and behaviors. We found that SI impacts the intention to adopt the platform. Thus, marketers could target social media, family, friends, and colleagues, to gain consumer’ engagement and influence consumer’ decision-making. Marketers might also target social engagement, such as advisements, billboards, and online threads. Marketers should target their consumers more specifically by starting with those around them.

Most importantly, this platform can increase the consumption of fruit and vegetables, especially organic varieties, thereby reducing fruit and vegetable waste (FVW). The platform might also increase the consumption of fruit and vegetables by providing easy and convenient access to these products.

## 7. Limitation and Future Research Directions

This study has limitations. By focusing on young adults, our findings may not be transferable to the general market. Based on the previous study, we chose not to focus on older age consumers because this group is not the ugly veggie target audience, which made this study ineffective. Additionally, older age adults may have limited Internet access. We believe that young adults are more likely to use the platform because of their flexibility and adaptability to new technology.

We were interested in separating the group into only three segments: low, moderate, and high health-conscious consumers. A further study may focus on another segmentation criterion using environmental consciousness level. In future work, perceived risk, creditability, and price sensitivity could be investigated to better understand consumer’ behavior.

## 8. Summary

Imperfectly shaped products contribute to FVW, even when they are nutritious and organic. An ugly veggies platform is an online food delivery concept aiming to address the needless waste of imperfectly shaped fruit and vegetables. We investigated market demand for an ugly veggie platform using the unified theory of acceptance and the use of technology (UTAUT) model. The study uses the UTAUT model to explore the relationship between various factors and the intention to use an ugly veggies e-commerce platform. The study is interested in studying the attitude of young adults towards imperfect fruit and vegetables because digital technology may impact their food choice. This study employed 390 young adult consumers. We classified those consumers as: (1) high; (2) moderate; and (3) low health-conscious consumers, using cluster analysis. Based on our findings, we conclude that PE and SI mainly impact consumer’ BI to use an ugly veggies platform.

By MGA, we found that FCs do not affect the BIs of any of the three consumer groups. However, PE was an important factor for highly health-conscious consumers. The moderately health-conscious consumer’ intentions are impacted by EE and SI. However, the low health-conscious consumers were not influenced by any tested factors. This research provides valuable insights into the factors influencing consumer’ behavioral intention to use an e-commerce platform for imperfect vegetables in Thailand. By understanding the impact of personal characteristics, health-consciousness, performance expectancy, facilitating conditions, and social influence, this study can inform the design and development of similar platforms, increasing the likelihood of success. Furthermore, this study has practical implications for stakeholders involved in the commercialization of ugly veggie products in Thailand, providing a deeper understanding of the needs and preferences of consumers in this market.

## Figures and Tables

**Table 1:** Details of constructs and factors.

Construct	Item	Observed Variables	Source
Performance expectancy	PE1	An ugly veggies platform may improve my performance in daily life.	[35,51,52]
	PE2	I expect that an ugly veggies platform saves time to purchase.	
	PE3	I expect to use an ugly veggies platform to buy healthy fruits and vegetables anywhere.	
	PE4	An ugly veggies platform will allow me to buy things more efficiently.	
Effort expectancy	EE1	I expect learning how to use an ugly veggies platform will be easy.	[35,51,52]
	EE2	An ugly veggies platform will have a user-friendly Web-App.	
	EE3	I will find an ugly veggies platform easy to use.	
	EE4	I expect it is easy to become skilled at using an ugly veggies platform.	
Facilitating condition	FC1	I expect to have the necessary resources to use an ugly veggies platform.	[35,51,52]
	FC2	I expect to have sufficient knowledge to use an ugly veggies platform.	
	FC3	There will be available assistance to help me with ugly veggies platform difficulties.	
	FC4	An ugly veggies platform will have a similar experience to other Internet services.	
Social influence	SI1	I expect people related to me will recommend using an ugly veggies platform.	[35,51,52]
	SI2	I believe that people influencing my behavior will suggest I use ugly veggies.	
	SI3	I will find that people using an ugly veggies platform will have more privileges.	
	SI4	I believe that my close people will suggest I use an ugly veggies platform.	
Behavioral intention	BI1	I intend to use an ugly veggies platform in the future.	[51,53]
	BI2	I intend to purchase vegetables on an ugly veggies platform.	
	BI3	I will find myself using an ugly veggies platform in daily life.	
	BI4	I plan to continue to use an ugly veggies platform frequently.	

**Table 2:** Details of the health-conscious measurements.

Construct	Item	Observed Variables	Source
Health-Conscious	HC1	I have the impression that I sacrifice a lot for my health.	[16]
	HC2	I think a good knowledge of how to eat healthily is important.	
	HC3	I continually ask myself whether something is good for me.	
	HC4	I think my food influences my health.	
	HC5	I am prepared to sacrifice things for my health.	
	HC6	My diet is well-balanced and healthy.	
	HC7	I am concerned about the quantity of food that I consume.	
	HC8	I take responsibility for the state of my health.	

All constructs of model adoption and consumer’ health-consciousness behavior measurement were designed on a 5-point scale (1 to 5 strongly disagree to strongly agree on the scale; higher values indicate greater consciousness about health [38]).

**Table 3:** The demographic profiles (descriptive statistics) of the study segments.

Demographic Variable	Categories	Segment 1 (High Health-Conscious)		Segment 2 (Moderate Health-Conscious)		Segment 3 (Low Health-Conscious)		Total		Chi-Square Test
		n	%	n	%	n	%	n	%	
Segment size		109	27.90	174	44.60	107	27.40	390	100	
Gender	Male	32	8.20	62	15.90	65	16.70	159	40.80	<0.001 **
	Female	77	19.70	112	28.70	42	10.80	231	59.20	
Marital Status	Single	102	26.20	170	43.60	105	26.90	377	96.70	0.105
	Married	7	1.80	4	1.00	2	0.50	13	3.30	
Family Size (person)	1	4	1.00	3	0.80	9	2.30	16	4.10	<0.001 **
	2	4	1.00	4	1.00	16	4.10	24	6.20	
	3	26	6.70	50	12.80	32	8.20	108	27.70	
	4	51	13.10	66	16.90	32	8.20	149	38.20	
	>4	25	6.40	50	12.80	18	4.60	93	23.80	
Age (years)	<25	36	9.20	141	36.20	34	8.70	211	54.10	<0.001 **
	25–34	73	18.70	33	8.50	73	18.70	179	45.90	
Income (THB)	<10,000	6	1.50	63	16.20	5	1.30	74	19.00	<0.001 **
	10,000–20,000	50	12.8	100	25.60	73	18.70	223	57.20	
	20,001–30,000	19	4.90	11	2.80	19	4.90	49	12.60	
	30,001–40,000	14	3.60	0	0.00	5	1.30	19	4.90	
	>40,001	20	5.10	0	0.00	5	1.30	25	6.40	
Educational Level	Diploma or below	13	3.30	18	4.60	8	2.10	39	10.00	0.012 *
	Bachelor	86	22.10	151	38.70	99	29.50	336	86.20	
	Master	8	2.10	5	1.30	0	0.00	13	3.30	
	Ph.D.	2	0.50	0	0.00	0	0.00	2	0.50	
Occupation	Student	5	1.30	159	40.80	31	7.90	195	50.00	<0.001 **
	Government Employee	2	0.50	15	3.80	30	7.70	47	12.10	
	State Enterprise Officer	7	1.80	0	0.00	6	1.50	13	3.30	
	Employee	58	14.90	0	0.00	34	8.70	92	23.60	
	Business owner	37	9.50	0	0.00	6	1.50	43	11.00	

Source: data adapted from authors (2023). Note: * denotes significance at <5%, ** denotes significance at <1%.

**Table 4:** Means and SDs classified by segments and Welch’s ANOVA test results.

Measure	Segment 1 (High Health-Conscious)		Segment 2 (Moderate Health-Conscious)		Segment 3 (Low Health-Conscious)		Welch’s Statistic	*p*-Value
	Mean	SD	Mean	SD	Mean	SD		
HC1	4.38	0.664	4.04	0.748	2.84	0.569	195.934	<0.001 **
HC2	4.38	0.635	4.21	0.641	2.36	0.65	340.236	<0.001 **
HC3	4.3	0.674	3.98	0.779	2.49	0.62	251.809	<0.001 **
HC4	4.44	0.645	4.18	0.672	2.37	0.541	431.770	<0.001 **
HC5	4.40	0.654	4.12	0.754	2.36	0.589	357.842	<0.001 **
HC6	4.32	0.665	3.99	0.79	2.41	0.531	337.171	<0.001 **
HC7	4.20	0.779	3.98	0.745	2.24	0.547	343.959	<0.001 **
HC8	4.25	0.747	4.05	0.715	2.39	0.611	281.387	<0.001 **

Source: data adapted from authors (2023). Note: HC = health-conscious, ** denotes significance at <1%.

**Table 5:** The goodness of fit of the measurement model.

Fit Index	Value	Threshold	Assessment
*p*-value	0.00		Acceptable
CMIN/df	1.739	<3.00	Passed
TLI	0.967	>0.90	Passed
CFI	0.974	>0.90	Passed
IFI	0.974	>0.90	Passed
RMSEA	0.044	<0.10	Passed

Source: data adapted from authors (2023). Note: TLI = Tucker–Lewis index; CFI = comparative fit index; IFI = incremental fit index; RMSEA = root mean square error approximation.

**Table 6:** Convergent validity.

Construct	Indicator	Loading	*p*-Value	Cronbach’s Alpha (Threshold = 0.70)	AVE (Threshold = 0.50)	CR (Threshold = 0.70)
Performance Expectancy (PE)	PE1	0.728	***	**0.807**	**0.513**	**0.808**
	PE2	0.708	***			
	PE3	0.691	***			
	PE4	0.736	***			
Effort Expectancy (EE)	EE1	0.772	***	**0.829**	**0.552**	**0.831**
	EE2	0.725	***			
	EE3	0.745	***			
	EE4	0.779	***			
Facilities Conditions (FCs)	FC1	0.762	***	**0.771**	**0.534**	**0.775**
	FC3	0.705	***			
	FC4	0.725	***			
Social Influence (SI)	SI1	0.771	***	**0.742**	**0.50**	**0.745**
	SI3	0.619	***			
	SI4	0.713	***			
Behavioral Intention (BI)	BI1	0.81	***	**0.829**	**0.616**	**0.828**
	BI3	0.806	***			
	BI4	0.736	***			

Source: data adapted from authors (2023). Note: AVE = average variance extracted; CR = composite validity; *** significant < 0.001.

**Table 7:** Discriminant validity.

HTMT Ratio Approach					
BI	-	-	-	-	-
SI	0.66	-	-	-	-
FCs	0.70	0.76	-	-	-
EE	0.68	0.68	0.81	-	-
PE	0.70	0.69	0.76	0.84	-

Source: data adapted from Authors (2023). Note: BI = behavioral intention; SI = social influence; FCs = facilitating conditions; EE = effort expectancy; PE = performance expectancy.

**Table 8:** The goodness of fit of the structural model (SEM).

FIT INDEX	Value	Threshold	Assessment
*p*-value	0.00		Acceptable
CMIN/df	1.739	<3.00	Passed
TLI	0.967	>0.90	Passed
CFI	0.974	>0.90	Passed
IFI	0.974	>0.90	Passed
RMSEA	0.044	<0.10	Passed

Source: data adapted from authors (2023). Note: TLI = Tucker–Lewis index; CFI = comparative fit index; IFI = incremental fit index; RMSEA = root mean square error approximation.

**Table 9:** Test results from the structural model.

Path	Relationships	Standardized Estimate	*p*-Value	Result
H1	PE > BI	0.271	0.028 *	Supported
H2	EE > BI	0.097	0.462	Rejected
H3	FC > BI	0.247	0.047 *	Supported
H4	SI > BI	0.230	0.024 *	Supported

Source: data adapted from authors (2023). Note: * significant < 0.05.

**Table 10:** Measurement invariance.

Fit Index	Configural Invariance	Metric Invariance	Scalar Invariance	Threshold
*p*-value	<0.001	<0.001	<0.001	
CMIN/df	1.63	1.637	1.646	<3.00
TLI	0.916	0.915	0.914	>0.90
CFI	0.925	0.922	0.918	>0.90
IFI	0.926	0.923	0.902	>0.90
RMSEA	0.04	0.041	0.041	<0.10
Assessment	Acceptable	Acceptable	Acceptable	

Source: data adapted from authors (2023). Note: TLI = Tucker–Lewis index; CFI = comparative fit index; IFI = incremental fit index; RMSEA = root mean square error approximation.

**Table 11:** The goodness of fit of the multigroup structural model.

Fit index	Value	Threshold	Assessment
*p*-value	0.00		Acceptable
CMIN/df	1.55	<3.00	Passed
TLI	0.926	>0.90	Passed
CFI	0.938	>0.90	Passed
IFI	0.939	>0.90	Passed
RMSEA	0.038	<0.10	Passed

Source: data adapted from authors (2023). Note: TLI = Tucker–Lewis index; CFI = comparative fit index; IFI = incremental fit index; RMSEA = root mean square error approximation.

**Table 12:** Test result from loading differences.

Path	Relationship	Low Health-Conscious		Moderate Health-Conscious		High Health-Conscious		Critical Ratio Difference			Threshold
		Std. Est.	*p*-Value	Std. Est.	*p*-Value	Std. Est.	*p*-Value	Low vs. High	Low vs. Moderate	High vs. Moderate	
H1	PE > BI	0.212	0.838	0.155	0.347	0.444	0.015 *	0.186	0.197	1.125	| 1.96 |
H2	EE > BI	−0.290	0.843	0.368	0.019 *	−0.223	0.296	−0.187	−0.216	−2.247 *	| 1.96 |
H3	FC > BI	−0.137	0.979	0.036	0.843	0.246	0.417	−0.084	−0.034	0.619	| 1.96 |
H4	SI > BI	0.300	0.891	0.370	0.015 *	0.317	0.308	−0.019	0.003	0.133	| 1.96 |

Source: data adapted from authors (2023). Note: PE = performance expectancy; EE = effort expectancy; FCs = facilitating conditions; SI = social influence; BI = behavioral intention; Std. Est. = standardized estimate; * = significant < 0.05.

## Data Availability

Data is contained within the article.

## Appendix A

**Questionnaire**

**Part 1**: Demographic Data of Respondents

1. Gender					
□ Male	□ Female				
2. Marital Status					
□ Single	□ Married				
3. Family Size	□ 1	□ 2	□ 3	□ 4	□ >4
4. Age					
□ Below 25 years	□ 25–34 years				
5. Income					
□ Below 10,000 THB	□ 10,001–20,000 THB	□ 20,001–30,000 THB			
□ 30,001–40,000 THB	□ Over 40,001 THB				
6. Level of education					
□ Diploma or below	□ Bachelor				
□ Master	□ PhD				
7. Occupation					
□ Student	□ Government Employee	□ State enterprise officer			
□ Employee	□ Business owner				

**Part 2**: Measurement items

1. Performance Expectancy (PE)

1.1. Ugly Veggies platform may improve my performance in daily life.

1.2. I expect that Ugly Veggies Platform saves time to purchase.

1.3. I will find Ugly veggies Platform can be used to buy healthy fruits and vegetables anywhere.

1.4. Ugly veggies platform will allow me to buy things more efficient.

2. Effort Expectancy (EE)

2.1. I expect that learning how to use Ugly veggies platform is easy for me.

2.2. Ugly Veggies Platform will have user friendly Web-App.

2.3. I will find that Ugly veggies Platform is easy to use.

2.4. I expect that it is easy to become skilled at using Ugly Veggies Platform.

3. Facilitating Condition (FC)

3.1. I expect that I have necessary resources to use Ugly Veggies Platform.

3.2. I expect that I have necessary knowledge to use Ugly Veggies Platform.

3.3. There will be available assistance to help me with Ugly Veggies Platform difficulties.

3.4. Ugly Veggies platform will have similar experience like other internet services.

4. Social Influence (SI)

4.1. I expect that people related to me will recommend using Ugly Veggies Platform.

4.2. I believe that people influencing my behavior will suggest me to use Ugly Veggies.

4.3. I will find that people using Ugly Veggies Platform will have more prestige in the circumstances.

4.4. I believe that my closed people will suggest me to use Ugly Veggies Platform.

5. Behavioral Intention (BI)

5.1. I intend to use Ugly Veggies Platform in the future.

5.2. I intend to purchase vegetables in Ugly Veggies Platform.

5.3. I will find myself to use Ugly Veggies platform in daily life.

5.4. I plan to continue to use Ugly Veggies Platform frequently.

6. Health consciousness measurement (HC)

Instruction: Consumer health consciousness behavior measurement designed on a 5-point scale (1 to 5 strongly disagree to strongly agree scale; higher values indicate greater consciousness about health [38].

6.1. I have the impression that I sacrifice a lot for my health.

6.2. I think a good knowledge of how to eat healthily is important.

6.3. I continually ask myself whether something is good for me.

6.4. I think my health is influenced by my food.

6.5. I consider that the deterioration of my health is very important.

6.6. I am prepared to sacrifice things for my health.

6.7. My diet is well-balanced and healthy.

6.8. I am concerned about the quantity of food that I consume.

6.9. I am usually aware of my health.

6.10. I take responsibility for the state of my health.
